# Controlling Biogenesis and Engineering of Exosomes to Inhibit Growth and Promote Death in Glioblastoma Multiforme

**DOI:** 10.3390/brainsci16020130

**Published:** 2026-01-25

**Authors:** Srikar Alapati, Swapan K. Ray

**Affiliations:** Department of Pathology, Microbiology, and Immunology, University of South Carolina School of Medicine, 6439 Garners Ferry Road, Columbia, SC 29209, USA; srikar.alapati@uscmed.sc.edu

**Keywords:** glioblastoma multiforme (GBM), therapy resistance, angiogenesis, autophagy, exosome biogenesis and engineering, apoptosis

## Abstract

Glioblastoma multiforme (GBM) is characterized by aggressive growth, extensive vascularization, high metabolic malleability, and a striking capacity for therapy resistance. Current treatments involve surgical resection and concomitant radiation therapy and chemotherapy, prolonging survival times marginally due to the therapy resistance that is built up by the tumor cells. A growing body of research has identified exosomes as critical enablers of therapy resistance. These nanoscale vesicles enable GBM cells to disseminate oncogenic proteins, nucleic acids, and lipids that collectively promote angiogenesis, maintain autophagy under metabolic pressure, and suppress apoptosis. As interest grows in targeting tumor communication networks, exosome-based therapeutic strategies have emerged as promising avenues for improving therapeutic outcomes in GBM. This review integrates current insights into two complementary therapeutic strategies: inhibiting exosome biogenesis and secretion, and engineering exosomes as precision vehicles for the delivery of anti-tumor molecular cargo. Key molecular regulators of exosome formation—including the endosomal sorting complex required for transport (ESCRT) machinery, tumor susceptibility gene 101 (TSG101) protein, ceramide-driven pathways, and Rab GTPases—govern the sorting and release of factors that enhance GBM survival. Targeting these pathways through pharmacological or genetic means has shown promise in suppressing angiogenic signaling, disrupting autophagic flux via modulation of autophagy-related gene (ATG) proteins, and sensitizing tumor cells to apoptosis by destabilizing mitochondria and associated survival networks. In parallel, advances in exosome engineering—encompassing siRNA loading, miRNA enrichment, and small-molecule drug packaging—offer new routes for delivering therapeutic agents across the blood–brain barrier with high cellular specificity. Engineered exosomes carrying anti-angiogenic, autophagy-inhibiting, or pro-apoptotic molecules can reprogram the tumor microenvironment and activate both the intrinsic mitochondrial and extrinsic ligand-mediated apoptotic pathways. Collectively, current evidence underscores the potential of strategically modulating endogenous exosome biogenesis and harnessing exogenous engineered therapeutic exosomes to interrupt the angiogenic and autophagic circuits that underpin therapy resistance, ultimately leading to the induction of apoptotic cell death in GBM.

## 1. Introduction

Gliomas are the most common primary brain tumors and arise from abnormal glial cells exhibiting unrestrained proliferation. The tumors mostly build up on astrocytes, star-shaped glial cells, which are vital to neuronal function, but gain an attribute of uncontrolled cell proliferation without cell differentiation. In addition to basic structural support, astrocytes are also involved in the formation and elimination of neuronal synapses, ionic homeostasis, the generation of neuronal rhythm and network patterns, and many more functions [1]. Thus, tumors built up on astrocytes prove to be detrimental to the patients, making treatments difficult and survival poor. Of the variations in glioma, glioblastoma multiforme (GBM) is the most common malignant brain tumor, as it accounts for 50.9% of all primary malignant brain tumors and 14.2% of all tumors [2]. GBM is by far the harshest and most aggressive of all the gliomas, with median survival times being low at around 8 months, and treatment with standard therapy only improves up to 21 months [3]. It is categorized as a grade IV brain tumor by the World Health Organization (WHO), which has also assigned classical biomarkers specific to GBM. These are predominant in adult populations over 50 years of age, exhibiting one of the three main variations in the isocitrate dehydrogenase (IDH) enzyme [2]. On that note, GBM contains the wild-type IDH enzyme, which lacks mutations on the IDH1, IDH2, and H3-3A genes like other variations in glioma [2]. Other common biomarkers in GBM are alterations seen on chromosomes 7 and 10, telomerase reverse transcriptase (TERT) promotors, and elevated epidermal growth factor receptor (EGFR) [4,5].

The current method of brain tumor treatment starts with surgical resection of the tumor. However, complete resection is never attainable since astrocytic gliomas spread around neurons, making their complete removal difficult without injuring these neurons. The focus during surgical resection has moved from completely removing the tumor to removing the tumor effectively and safely [6,7]. After surgery, radiation therapy and concomitant chemotherapy are used to enhance survival and protect neurological function [6]. Radiation therapy has shown to be vital in improving survivability in GBM and can be detrimental if not given after surgery. Chemotherapeutics are then used during radiation therapy and after it as maintenance treatment. There are a variety of drugs used as chemotherapeutics, but the current gold standard is temozolomide (TMZ) [8]. TMZ is a small, lipophilic alkylating agent. This lipophilic property allows it to freely cross the blood–brain barrier (BBB), which is a huge problem most of the drugs face during treatment of GBM and other brain tumors, as the BBB prevents 98% of drugs from entering the brain [9]. TMZ is taken orally, and its peak plasma concentration is reached at one hour. At lower pHs, TMZ is stable and non-functional; at pHs higher than seven, TMZ is hydrolyzed and activated to 3-methyl-(triazen-1-yl) imidazole-4-carboxamid (MITC) and is functional as an alkylating agent. Since physiological pH is naturally above seven, hydrolyzation occurs rapidly and leads to a shorter half-life of MITC, right under two hours, and is completely cleared from the plasma after 8 h [7].

As an alkylating agent, TMZ specifically methylates DNA at the O6 guanine, N7 guanine, and N3 adenine residues. These modifications cause the guanines to pair with thymine instead of cytosine during transcription, resulting in cell cycle arrest at G2/M phase and eventually cell death because of single-strand and double-strand DNA breakage [10]. Although TMZ is successful in methylating DNA, there are various DNA repair mechanisms that recognize this DNA damage and correct it, resulting in chemotherapeutic resistance. The O6-methylguanine-DNA methyltransferase (MGMT) enzyme is one of the enzymes responsible for DNA repair, as it specifically removes the methyl group from the O6 guanine that TMZ has put on. Therefore, higher MGMT levels lead to higher TMZ resistance and lower survivability of the patients [6]. Mismatch repair (MMR) is another mechanism that repairs DNA by excising the incorrect base match from the newly synthesized DNA strand. Mutations in MMR genes are seen in GBM, which impairs the ability of MMR to repair, and thus, another form of therapy resistance occurs [10]. The last DNA repair mechanism that is exploited by the tumor cells for chemotherapy resistance against alkylating agents is base excision repair (BER). This mechanism targets the methylation of N7 guanine and N3 adenine residues and repairs DNA using a multitude of proteins, making mutations in this mechanism another way GBM cells can become therapy resistant to TMZ [9].

Yet another mechanism of resistance to TMZ is the induction of autophagy instead of apoptosis, which is the gold standard and the most desirable cell death mechanism following tumor treatment. Autophagy is used by cells to maintain homeostasis and recycle cellular materials. It is triggered by various conditions such as starvation, hypoxia, toxic agents, and DNA damage [11]. Since TMZ damages DNA through guanine methylation, studies show that autophagy is induced, allowing tumor cells to reuse material and continue to grow, making treatment ineffective. Therapeutics for targeting autophagy have been studied for cancer treatment, as this is a major mechanism used by the cells for tumor growth. Multiple signaling pathways can lead to autophagy, making it difficult to understand which pathway is being used by the tumors. One crucial pathway tumors use is the inhibition of mechanistic target of rapamycin (mTOR) by 5′ adenosine monophosphate-activated protein kinase (AMPK) in the initiation step, allowing Unc-51-like kinase 1 (ULK1) to phosphorylate a complex composed of Beclin-1 and vacuolar sorting protein 34 (Beclin-1/VPS34) in the nucleation step, causing induction of autophagy [12]. Activation of AMPK is another mechanism of TMZ, causing more resistance to being built up, making it vital to study other methods of inhibition for autophagy.

There are many other drugs in place for using in treatment of GBM or in conjunction with TMZ to combat the resistance that is built up. Many are currently in clinical trials and target other common biomarkers of GBM. Common EGFR tyrosine kinase inhibitors (TKIs) are gefitinib, dacomitinib, osimertinib, and nimotuzumab. While in vitro studies show decreased tumor growth, clinical trials have proven them to be not as effective due to poor penetration through the BBB. Other common drugs, such as cabozantinib, which is a mesenchymal–epithelial transition (MET) factor, and vascular endothelial growth factor receptor 2 (VEGFR2) inhibitor, bevacizumab (an anti-VEGF antibody), and bortezomib (a proteosome inhibitor), all encounter the same problem of crossing the BBB [13]. Therefore, alternatives to TMZ have been limited in usage for GBM chemotherapy by the BBB, which is crucial factor contributing to therapy resistance that keeps building up.

The current method of crossing the BBB is through passive diffusion [14]. The lipophilic nature of alkylating agents, such as TMZ, allows for the drug to diffuse across the cell membrane. However, this diffusion is limited, and further modifications to decrease charge and hydrogen-bonding capabilities are being worked on to increase the lipophilic nature to increase permeability [15]. Another approach is to improve the half-life of these drugs by factors such as cerebral blood flow, surrounding tissue degradation, and other extracellular pathways. With the goal of increasing exposure of the drug to the BBB, these factors play an important hurdle in improving drug efficacy for central nervous system (CNS) tumors [14]. The difficulty in finding the right balance in modifications has led to another approach of disrupting the BBB itself. Through various techniques of osmotic disruption, focused ultrasound, high-intensity ultrasound, and electromagnetic radiation have all been tried. These approaches, however, have found unwanted side effects, such as excessive heat generation, non-specific tissue damage, chronic pain, intense inflammation, and even the development of chronic diseases [6,14].

The most recent approach goes back to improving the passive diffusion of these drugs through nanodelivery systems. These are small molecules that can be modified to mimic surrounding cells, bind to receptors, or store drugs for later release. Liposomes, micelles, nanocapsules, and peptide-based nanoparticles are some of the more prominent methods of delivery [16,17,18]. Liposomes have been studied the most and have been used in other areas of the body. However, they have been shown to have rapid clearance through the reticuloendothelial system and accumulate in the liver and spleen instead of reaching the tumor site in the brain. They also trigger a complement activation-related pseudoallergy (nonallergic hypersensitivity reaction due to drug treatment), leading to the release of histamine, tryptase, and leukotrienes [16,19]. Both limitations reduce the exposure time and thus are not effective delivery options.

**Figure 1:**
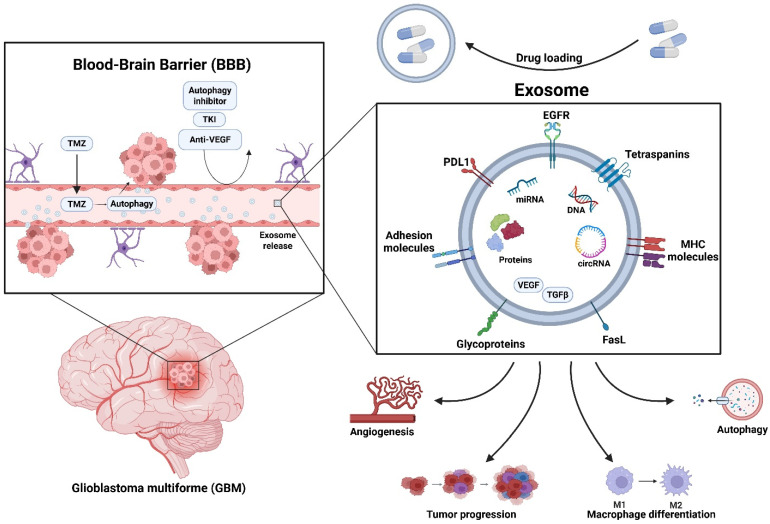
Functions of exosomes within the GBM tumor microenvironment to promote therapy resistance and tumor growth. These are also potential natural nanovehicles for drug delivery to the tumor.

This brings us to the emerging and alternative option of drug delivery using extracellular vesicles (EVs). EVs, especially exosomes, are a current hotspot in the drug delivery community and provide a promising alternative to combat the difficulties in crossing the BBB [16,20,21,22,23]. While they are like liposomes, the key difference is that they are made endogenously by all cell types, versus the synthetically made liposome [23,24,25]. Exosome biogenesis is mediated by multiple molecular machineries, including the endosomal sorting complex required for transport (ESCRT), Rab GTPases, and other trafficking proteins that regulate vesicle formation, specific cargo selection and loading, and secretion. Interestingly, this molecular process intersects extensively with the autophagic process through shared vesicular intermediates, providing a link between autophagy and exosome-mediated signaling in GBM. It has been found that exosomes are abundant in tumor microenvironments and are vital to the progression and proliferation of the tumor. Their cargo can vary drastically, containing growth factors contributing to angiogenesis, microRNAs (miRNAs) that modify specific genes to aid in tumor survivability, and many other materials. A current hotspot in exosome research focuses on how miRNA-210 contributes to the maintenance of the hypoxic tumor microenvironment that promotes the progression of cancers [26]. Another area of interesting research includes utilizing exosomes as therapy delivery vehicles after isolating them and then loading them with therapeutics to make engineered exosomes to target specific sites in the body. Exosome engineering can be controlled and utilized to improve the effectiveness of therapeutics to cross the BBB or to induce other unique pathways, such as current work performed to induce ferroptosis in GBM via engineered exosomes [27] (Figure 1).

In this review, we critically examine all the effects exosomes have on GBM—the good and the bad. We go into exosome biogenesis and composition to have a better understanding of what makes them so valuable to cancers and how they are key players in the development of therapy resistance mechanisms. Subsequently, we go through current methods of exploiting exosomes by either inhibiting their endogenous biogenesis or exogenous engineering them as drug delivery vehicles to treat GBM.

## 2. EVs as Crucial Players in Cell Communications and Functions

To increase the delivery of therapeutics across the BBB, a huge focus on nanodelivery methods has arisen. Of all the options, EVs possess the potential for great efficacy in drug delivery, especially for the stubborn GBM tumors. In general, EVs are used and secreted by all types of cells for cell-to-cell communication and material delivery, such as proteins, lipids, nucleic acids, mRNA, tRNA, coding and noncoding RNAs, miRNAs, extracellular DNA, and even organelles. EVs can even be seen being used during embryonic development by embryonic stem cells for communicating with each other when deciding differentiation or self-renewal [21]. They are composed of a phospholipid bilayer with various receptors, major histocompatibility complex (MHC) molecules, and tetraspanins [16]. Based on the cell of origin, the composition of the lipid membrane, receptors, and cargo varies greatly. For example, EVs derived from GBM cells have been shown to carry the EGFR variant III (EGFRvIII), which is generated due to the mutation of EGFR. This mutation of EGFR deletes its ligand-binding domain, causing it to be constitutively active, and allowing it for tumor cells to continuously grow [16].

With each EV containing a specific set of receptor types and cargo, they can be considered as a fingerprint of their parental cells; thus, they can be used as biomarkers for specific disease states [28]. In GBM patients, there is a 5.5-fold increase in circulating EVs in plasma circulation when compared to healthy donors, showing how vital these are in tumor survival and growth [29]. There is currently no specific correlation between the molecular markers typically tested for GBM and the receptors on the EVs found; however, characterization of the protein cargo has been shown to include eleven common proteins such as vWF (von Willebrand factor), APCS (Amyloid P component, serum), C4B (Complement component 4B), AMBP (Alpha-1-microglobulin/bikunin precursor), APOD (Apolipoprotein D), AZGP1 (Alpha-2-glycoprotein 1, zinc-binding), C4BPB (C4b-binding protein beta chain), Serpin3 (Serine protease inhibitor), FTL (Ferritin light chain), C3 (Complement component 3), and APOE (Apolipoprotein E) found in the coagulation cascade and regulators of iron metabolism [30]. Based on these characteristics listed above, EVs show a promising avenue for developing drug delivery systems. Since they are produced by endogenous cells, they can bypass common immune surveillance mechanisms as they can be recognized with some surface proteins such as CD47 (Cluster of Differentiation 47 that gives ‘do not eat me’ signal to macrophages) [24]. This greatly increases their stability in plasma circulation and gives greater opportunity for the penetration of barriers. In the case of GBM, the BBB is of immense focus as it limits the efficacy of 98% of small-molecule drugs. Although there has been solid evidence that EVs can cross this barrier, the mechanism is not very well-understood. However, with the vast number of receptors and receptor types, these vehicles can have a considerable number of receptor–ligand interactions when compared to other synthetic methods of delivery and can be a contributing factor to their superior ability to cross the BBB. These interactions lead to their rapid uptake through varying endogenous endocytosis mechanisms based on their parental cell, particularly at lower pHs [16].

Another important benefit of EVs is their low toxicity as drug delivery vehicles. Since cells naturally produce these, they have minimal reactions with the immune system and thus have no adverse effects. Several preclinical and clinical trials are currently testing the cytotoxic, if any, effects that the modified EVs may have. While results are still in the initial stages, it is evident that the EVs from parental origin pose no or negligible risk. For example, engineered EVs from the wild-type cell line Expi293F in BALB/c mice over 24 h showed no notable toxicity or immune response [31]. Tumor cell-derived EVs show clinical benefits such as potential for noninvasive and targeted treatment; however, their standardization, reproducibility, and immune interaction are still tough challenges that need to be addressed [32]. Further work needs to be performed to fully investigate these and eliminate potential risks, but overall, current understandings suggest that EVs can be used as a safe alternative to other nanodelivery vehicles for drug delivery to the cells, including tumor cells [16]. Of all the EV types, exosomes have so far shown the greatest potential for being exogenously engineered for drug delivery to GBM cells.

### 2.1. Exosomes

EVs can be distinguished into three main types: microvesicles, apoptotic bodies, and exosomes. The primary way to distinguish these is based on their formation and size. Microvesicles and apoptotic bodies are formed from the outward budding of the plasma membrane, producing a heterogeneous population of vesicles ranging from 50 to 1000 nm in size. Apoptotic bodies are specifically generated from the apoptotic death mechanism during cell fragmentation. Contrastingly, exosomes are produced by the inward budding of the plasma membrane forming early endosomes [23]. ESCRT protein complexes are then used to mature the early endosomes into late endosomes or multivesicular bodies (MVBs) [33]. ESCRT are vital to the production of exosomes and can increase or decrease exosome secretion as they are used to make small intraluminal vesicles (ILV) inside these MVBs. Transmembrane protein tetraspanins, such as CD9, CD8, CD63, Tspan8, and small integral membrane proteins of the lysosome/late endosome (SIMPLE), are also known to be involved in exosome release and can act independently of the ESCRT protein complex to create MVBs and ILVs [34].

The MVBs that are produced have two main choices for the next step: directed to lysosomes or the plasma membrane. The mechanism for lysosomal degradation has not been completely discovered; however, it is thought that the MVBs undergo ISGylation, which is a method of protein regulation involving the attachment of a small protein tag, called interferon-stimulated gene 15 (ISG15), to other proteins inside the cells. In essence, ISGYlation is a post-translational ubiquination-like protein modification that ultimately causes the MVBs to fuse with the lysosome and be broken down [35]. Induction of ISGylation has shown to impair exosome secretion overall, confirming that this marks MVBs for degradation. For the secretion pathway, several protein interactions must occur. Actin and its association with the microtubule cytoskeleton are vital for this process, with actin binding protein cortactin being used for trafficking and docking of MVBs. Regulation of cortactin is performed by Rab GTPases, although the full mechanism is not yet known. Since this process also involves proteins, like synaptotagmin and calmodulin, that are Ca^2+^ sensitive, increased Ca^2+^ levels have shown an increase in exosome release [36].

Once MVBs reach the plasma membrane, they encounter an energy barrier when fusing with the plasma membrane that is overcome by tethering factors, Rab and Ras GTPases, and soluble *N*-ethylmaleimide-sensitive factor attachment protein receptors (SNAREs) [37]. SNAREs can be classified as R-SNAREs or Q-SNAREs, with fusion typically involving one R-SNARE and three Q-SNAREs. Knockdown or induction of any one of these fusion factors can result in an increase or decrease in exosome release, mainly depending on the parental cell. Another method of decreasing the energy barrier of fusion involved increasing the levels of cellular ether lipids, as the lipid–protein interactions help reduce the energy barrier. Once the MVBs have successfully fused with the plasma membrane, the ILVs inside become the exosomes that are released into the extracellular environment [23,38]. The process of endogenous exosome formation or biogenesis is important to understand as each of these steps can be targeted to either inhibit the formation of exosomes or to load exogenous drugs for delivery of therapy to the target cells.

### 2.2. Exosome Isolation

Since the use of exosomes as a delivery vehicle is relatively new, a major barrier for preclinical or clinical use is finding the most efficient way to isolate them from a cell culture [39]. The main methods of exosome isolation are ultracentrifugation, size-exclusion chromatography, polymer-based precipitation, affinity capture, and microfluidics. Each method results in exosomes of different concentrations, sizes, and purities [40]. The current gold standard for exosome isolation is differential ultracentrifugation [41]. This process involves multiple steps, changing the centrifugal speeds from low to high to filter out larger EVs and particles to result in the smaller exosomes. Increased centrifuge time has shown increased exosome selectivity; however, longer time periods can lead to mechanical damage to the exosome membrane and soluble protein contamination. The main benefit of this method is that it requires little technical expertise and is relatively inexpensive. Yet, the lengthy process, high starting volume, and lack of specificity are drawbacks to this method and require it to be used in combination with other techniques to increase the yield and purity [42].

### 2.3. Exosome Cargo Loading Techniques

There have been many recent developments in the loading and modification of therapeutic exosomes [43]. Loading exosomes with the desired cargo can be thought of in two separate ways: endogenously and exogenously. The endogenous pathway occurs during the normal formation of exosomes. Cargo can be loaded into exosomes in many ways, starting with the first inward budding of the plasma membrane, making the early endosome, and extracellular material can be taken up that can be a part of the eventual exosome [44]. Once the late autophagosome or MVB forms, cellular material from the cytosol can also be taken up. Using ESCRT and other accessory proteins, this cargo, namely proteins, can be sorted into and out of the MVBs. For the diverse types of RNAs, no common mechanism of loading is currently known; however, studies show that miRNAs are loaded using RNA-binding proteins that bind to specific miRNAs [45]. Induction and inhibition of these sorting and binding proteins offer opportunities for treatment for GBM, as exosomes prove to be vital in therapy resistance.

Exogenous loading techniques are performed after isolating exosomes [46,47,48]. Although exosomes are amphiphilic in nature, hydrophobic compounds are more likely to be passively diffused through the exosome membrane. Modifying the membrane with increasing levels of cholesterol increases the number of hydrophilic compounds that enter, and can be a potential strategy. In addition to the concentration gradient-dependent diffusion, other methods involve creating pores in the exosome membrane to allow compounds to move freely. Sonication and electroporation are two of the main methods to achieve these pores and lead to the exosomes growing, but are limited in clinical settings because of aggregations of RNA. Extrusion and chemical methods, such as saponin dialysis for improving encapsulation of cargo into the exosomes, have also been used. These exogenous loading techniques are widely employed for utilization of exosomes as drug delivery vehicles for the treatment of GBM [45].

Beyond cargo loading, the stability of exosomes is vital to their function as a delivery vehicle, whether they are endogenously produced by the tumor cells to promote survival or exogenously engineered to carry therapeutic cargo. Studies about the post-secretion kinetics of tumor-derived exosomes show that there is a short terminal half-life through circulation, minutes to an hour, by rapid clearance by the reticuloendothelial system [49]. This rapid clearance reflects efficient cellular uptake rather than instability. Along with the lipid bilayer and other traits, such as various surface proteins stabilizing structure and deterring enzymatic degradations, exosomes are now considered to be the outstanding delivery vehicles [50].

## 3. Exosomes Play Critical Roles in Therapy Resistance in GBM

Treatment of GBM has been proven to be challenging due to the aggressive nature of this tumor and its adaptability to various therapeutics to confer mechanisms of therapy resistance. Therapy resistance in GBM can be looked at as focusing on chemotherapeutic drug resistance or tumor invasion. Both are thought to be exemplified by exosomes within the tumor microenvironment (TME), as exosomes can transfer a wide variety of molecules such as enzymes, receptors, DNA, noncoding RNAs (ncRNAs), and proteins [21,22]. Although the therapy resistance factors are classically considered to originate from direct secretion by the tumor cells, increasing evidence indicates that a substantial fraction is packaged and disseminated through tumor-derived exosomes. While the precise proportion of growth factors and regulatory molecules delivered via exosomes versus free soluble secretion remains unclear, exosomes have been shown to function as effective intercellular delivery vehicles and are markedly increased in patients with malignancies such as GBM [51].

A common trope found in GBM cells is the constitutively active EGFRvIII protein, which is found heavily in GBM-derived exosomes [52]. Around 30 to 50 percent of GBM mutations have the amplification of this in-frame deletion that causes the tyrosine kinase domain of this protein to be active, leading to activation of the signaling cascades like Akt and mitogen-activated protein kinase (MAPK) pathway that cause cellular proliferation, migration, and invasion of tumor cells. Typically, tyrosine kinase inhibitors (TKIs) would be the option for treatment as a form of inhibition. A longitudinal observational study looked at GBM patients who were treated with dacomitinib, a TKI, and showed that it was not effective in most patients; for those who had success with dacomitinib, their progression of GBM diminished drastically, with some lasting over a year progression-free [53]. Studies were performed on identifying RNA signatures in the EVs of these patients to use as biomarkers, of which TKIs could work on best. It was found that eight genes were commonly found in EVs of the patients who responded to TKI treatment and thirteen genes for those who did not respond [53]. Interestingly, EV content can also be modified upon oncogenic mutations and can expand the therapy resistance. One study compared the compositions of GBM EVs with and without the mutation and found that there were up to 254 proteins (24%) that were significantly affected when they had EGFRvIII activation [52]. These proteins were associated with promoting focal adhesion, cell junction, cell adhesion, and invasion, all of which are important for tumor proliferation. These studies show how understanding the composition of GBM exosomes has helped investigators recognize the methods of therapy resistance they cause.

### 3.1. Angiogenesis in Conferring Therapy Resistance in GBM

Another hallmark of GBM is the induction of angiogenesis, the formation of new blood vessels from existing ones. This is exemplified in GBM, as many pathways are dysregulated to maintain and grow tumors. As an increase in blood flow to the tumor site gives the tumor cells the nutrients and oxygen they need to proliferate and invade, this process requires therapeutic interventions in GBM [54,55,56]. GBM-derived EVs have been shown to contain a variety of different pro-angiogenic and anti-angiogenic factors. Of note, they contain VEGF and transforming growth factor β (TGFβ). VEGF, the origin of which is located on the short arm of chromosome 6, is crucial and one of the most predominant factors in promoting angiogenesis. This is upregulated in hypoxic conditions, which are prevalent in the TME of GBM as the intake of oxygen from the growing tumor cells exceeds the input to the body. The VEGF family can bind to three tyrosine kinase receptors: VEGFR1, VEGFR2, and VEGFR3. Each of these has multiple modes of signaling pathways, such as protein kinase C (PKC)/extracellular regulated protein kinase (ERK), focal adhesion kinase (FAK), phosphoinositide 3-kinase (PI3K)/Akt, and Src kinases, all of which, in turn, produce angiogenic effects [57]. Bevacizumab, a humanized monoclonal antibody specific to VEGF-A, is the first Unites States Food and Drug Administration (FDA)-approved therapy to be used in a clinic for GBM treatment to inhibit angiogenesis [58]. This isoform is targeted as it has been found to be upregulated in GBM tissues in clinical settings as well as in cell lines. Exosomes derived from these tissues were found to have significantly increased expression of VEGF in the exosomes themselves, and silencing of VEGF-A resulted in an increased portion of cells being arrested in the G0/G1 phase and decreased portions in the S phase, indicating cell cycle arrest, and decreased migration and invasion [59]. The VEGF-C isoform has also been found in GBM-derived exosomes and is known to drive the formation of lymphatic vessels along with angiogenesis. The binding of VEGF-C to VEGFR2 showed downregulation of the Hippo signaling cascade, activating transcription factors Yes-associated protein (YAP) and tafazzin (TAZ, phospholipid-lysophospholipid transacylase needed for maturation and remodeling of cardiolipin in the inner-mitochondrial membrane) ultimately leading to cell growth and proliferation [60,61].

**Figure 2:**
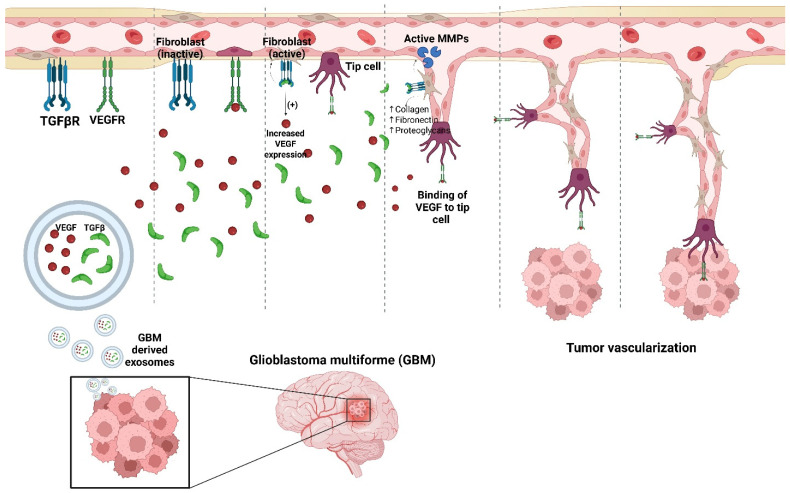
Overexpression of pro-angiogenic factors in GBM-derived exosomes results in increased angiogenesis and overall tumor vascularization. Factors VEGF and TGFβ are known to be vital in the formation of new vessels, with VEGF leading the tip cell and TGFβ activating matrix metallo proteinases (MMPs) for basement membrane degradation and extracellular matrix crosslinking through activated fibroblasts releasing collagen, fibronectin, and proteoglycans. Vascularization of GBM results in a worse prognosis and is a vital way GBM tumors evade treatment and confer therapy resistance.

As mentioned above, another common pro-angiogenic factor is TGFβ. Along with other cellular processes, such as proliferation, metabolism, motility, and migration, TGFβ is key to tumor growth, as it interacts with many different signaling cascades, with the suppressor of mother against decapentaplegic (SMAD) family proteins playing a critical role in connecting them to each other. TGFβ has interesting properties in tumor progression as it inhibits proliferation and acts as a tumor suppressor during the initial stages of tumorigenesis but switches to a pro-tumor factor during late stages [59]. Upon binding to the TGFβR, TGFβ acts as a pro-angiogenic factor by upregulating VEGF. Inhibition of TGFβ in oral squamous cell carcinoma resulted in decreased VEGF expression, showing that they are related, yet the mechanism of how is not completely understood [62]. In GBM, the TGFβ1 isoform is found in exosomes for the progression of the tumor, and when knocked down, they induce apoptosis, decreased migration, and decreased proliferation [59]. TGFβ receptors are also susceptible to inhibition by various molecules, inhibiting the angiogenic pathway. GBM-derived exosomes resulted in increased angiogenesis and overall tumor vascularization (Figure 2). One example shows the GBM-derived exosomes having an elevated amount of circARID1A, a circular RNA (circRNA) directly bound to miR-370-3p (a miRNA that inhibits TGFβRII), because circARID1A sponges or inhibits the effects of miR-370-3p, allowing TGFβ to bind to its receptors and promote the tumor growth pathways [63]. Inhibition of circARID1A showed significant decreases in migration and growth. This goes to show the complexities in the TGFβ signaling pathway and its prominence in angiogenesis and therapy resistance in GBM.

### 3.2. miRNAs in Conferring Therapy Resistance in GBM

miRNAs are short, 18–22 nucleotide-long noncoding RNAs (ncRNAs) that are prominent in the cargo of exosomes. These sequences are powerful gene expression regulators at the post-transcription level, with each capable of targeting hundreds of different mRNAs. Studies have established that miRNAs participate in many normal biological processes like cell cycle regulation, apoptosis, proliferation, and differentiation. However, many of the miRNAs are dysregulated, leading to events that help growth and therapy resistance in cancer cells [64]. Therapeutic modulation of the expression of miRNAs proved to be a promising avenue for overcoming therapy resistance and controlling GBM growth [65,66,67]. Investigations revealed a respectable number of miRNAs commonly occurring in GBM and the molecular mechanisms promoting therapy resistance (Table 1). Some miRNAs acting as tumor promoters (onco-miRs) are upregulated, whereas others acting as tumor suppressors (TS-miRs) are typically inhibited or found at low levels in cancers. In GBM tissues, the tumor promoter miR-191 is upregulated for targeting the transcripts of the N-deacetylase and N-sulfotransferase 1 (NDST1) gene, the lower expression of which correlates with poorer patient survival [68], whereas the tumor suppressor miR-200c, which targets transcripts of the Zinc finger E-box Binding homeobox 1 (ZEB1) gene that plays a role in epithelial–mesenchymal transition (EMT) and cancer progression, is found to be downregulated by EGFR to diminish its tumor-suppressing effects [69].

One of the most abundant miRNAs found is miR-21, the first onco-miR, which acts to cause oncogenesis. The targets of this onco-miR are vast, as it upregulates certain genes and downregulates others. Composition of GBM exosomes has been examined by numerous studies, all showing abundance of miR-21 and its effects on tumor progression. One of its targets is the signal transducer and activator of transcription 3 (STAT3) and stem cell markers (e.g., Oct4, Wnt, Sox2, and Nestin) that trigger signaling cascades for VEGF, as well as promoting binding of VEGF to its receptors [75]. Use of STAT3 inhibitors (e.g., pacritinib) has shown to decrease colony formation and decrease the ability to generate M2 macrophages, which secrete tumor-promoting factors. Other studies show that inhibitory effects of miR-21 are targeted towards programmed cell death 4 (PDCD4), human MutS homologue 2 (hMSH2), and phosphatase and tensin homolog (PTEN), all of which are well-known tumor suppressors [73]. miR-374b-3p found in GBM exosomes also downregulates PTEN, in turn, leading to the development of M2 macrophages and the release of the vascular cytokine TGFβ and the cytokine activator matrix metallopeptidase-9 (MMP-9) [77]. miRNAs have also been found to target genes that control the expression of proteins. For example, miR-9, which upregulates multidrug resistance 1 (MDR1), leading to an increase in the P-glycoprotein (P-gp) cell surface protein, contributes to therapy resistance to TMZ [77].

As discussed above, TMZ resistance is acquired in part due to DNA damage repair mechanisms being upregulated. Some miRNAs have been found to be responsible for the control of the expression of these proteins. For example, miR-151a specifically inhibits X-ray repair cross-complementing protein 4 (XRCC4), which contributes to the non-homologous end joining (NHEJ) mechanism of DNA damage repair. GBM exosomal composition studies show that miR-151a is expressed at extremely low levels, leading to more non-homologous repair after TMZ administration, leading to chemoresistance [84]. Focus on using anti-miRNA strands to inhibit these onco-miRs has been shown to improve sensitivity to TMZ and diminish the proliferation, migration, and invasion of GBM tumors.

Another miRNA commonly found in GBM exosomes is miR-451. In some studies, it has been shown to be found in numerous amounts, while in others it is severely lowered. One study has shown that its effects can vary with its levels. Upregulation of miR-451 results in downregulation of Ca^2+^-binding protein 39 (CAB39), in turn, upregulating the AMPK/mTOR pathway that promotes cell proliferation. miR-451 also inhibits Rac1 (a small, about 21 kDa, signaling G protein or a GTPase), which promotes the formation of lamellipodia (leading edge at the front of the cell) and cell migration. Lower levels of miR-451 in exosomes showed that there was increased cell migration [85]. This effectively showed that depending on their levels, miRNAs could have different effects on tumor cells and pose another challenge when targeting them as therapeutics.

### 3.3. Autophagy in the Context of GBM

The process of autophagy has been extensively studied, as it is a vital process that all cancers, including GBM, utilize to increase survivability and cell proliferation [86,87]. Since TME of GBM is extremely hypoxic in nature, autophagy runs rampant, and it aids the success of tumor growth [88,89]. Exosomes have been found to aid the induction of autophagy with the wide variety of cargo they carry. A complex triad of exosomes, autophagy, and chemoresistance is highly likely to contribute to cancer progression [90]. Exosome function and autophagy induction run in parallel, using much of the same cellular machinery, which is useful for understanding development of therapy resistance in GBM (Table 2).

#### 3.3.1. Exosomes Use Multiple Mechanisms to Induce Autophagy in GBM

Exosomes contain cargoes that have a host of potential effects. Tumor-derived exosomes are studied extensively in their composition and their effects to see how they contribute to tumor cell proliferation. For example, a study of exosomes derived from human bronchial epithelial cells showed the mechanistic effects of miR-7-5p [96]. Studies show that miR-7-5p directly regulates the EGFR/Akt/mTOR pathway, leading to further increase in induction of autophagy [97]. Since GBM-derived exosomes are known to carry EGFR protein, this could be a vital way for attenuation of the autophagy response. miR-7-5p has been characterized in GBM tumor-derived exosomes; although it inhibits a growth factor, miR-7-5p increases the autophagy response, which is a focus of therapy resistance [98]. GBM exosomes also carry programmed death-ligand 1 (PD-L1), which activates AMPK. AMPK then activates ULK1 (the mammalian homolog of autophagy-related gene 1 or ATG1 in yeast), a prominent protein in the autophagy pathway, to start the formation of the autophagosome. This kinase also phosphorylates mTOR complex (mTORC) to prevent it from its inhibitory action on ULK1. Phosphorylation event by PD-L1 activated AMPK, leading to increased levels of autophagy, and use of the autophagy inhibitor 3-methyladenine (3-MA) confirmed that autophagy was the mechanism of TMZ resistance in GBM [99]. Hypoxic GBM-derived exosomes indicated elevated levels of miR-155-3p, which directly inhibited the cyclic AMP-responsive element binding protein 3 (CREB3) regulatory factor (CREBRF), which inhibits CREB3. CREB3 is associated with ATG5 in autophagy formation, making miR-155-3p inhibition of CREB3 a method of inducing autophagy. This same study found higher levels of interleukin-6 (IL-6), which increased levels of miR-155-3p and activated the STAT3 pathway, promoting M2 macrophage polarization. M2 macrophages are anti-inflammatory and release anti-inflammatory signals, such as IL-10 and TGFβ, aiding tumor in evasion of the immune system surveillance and thus reducing apoptotic killing of tumor cells [100]. High level expression of PD-L1 is also seen in GBM [101].

#### 3.3.2. Exosomes and Autophagy Connection

**Figure 3:**
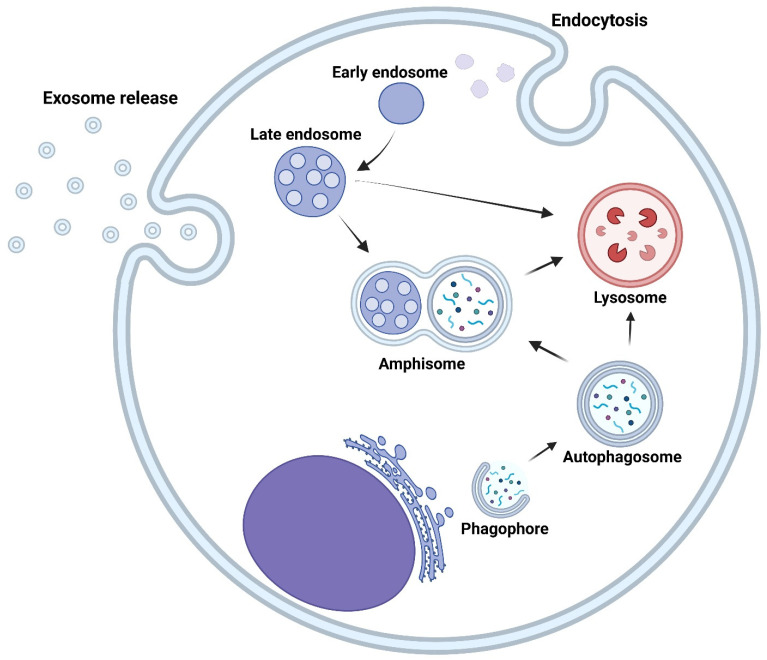
The interconnected machinery of autophagy and exosome production. Both processes converge at the amphisome, an organelle formed from the fusion of the autophagosome and late endosome.

The cellular mechanism of autophagy and exosome formation runs in parallel, utilizing much of the same machinery (Figure 3). Most commonly, macroautophagy occurs when cellular content is sent to autophagosomes that combine with lysosomes for recycling damaged materials in the cells. Other degradative autophagy types include microautophagy and mitophagy. Secretory autophagy is a non-degradative autophagy pathway that is most like exosome release [102,103]. The main intersection between the two pathways is the amphisome, a unique organelle created with the fusion of autophagosomes and multivesicular bodies (MVBs, a special kind of late endosomes). Here, the ESCRT sorting complex targets the cargo in the amphisome for the creation of ILVs in MVBs for exosome secretion or ubiquination of proteins for lysosomal degradation. Many experiments have been performed regarding the connection between autophagy and exosomes using ESCRT studies; since both processes converge at the amphisome, inhibiting ESCRT machinery is highly likely to provide an attractive new avenue for GBM therapies, as it results in decreased levels of both autophagy and exosomes that contribute to tumor survival and growth.

One study using neuronal cells looked specifically at hepatocyte growth factor-regulated tyrosine kinase (Hgs) on ESCRT-0, the element that recognizes ubiquination. Silencing ESCRT-0/Hgs resulted in the failure of autophagosomes to fuse to lysosomes to form the amphisome, resulting in decreased autophagy [104]. Silencing of ESCRT-0/Hgs resulted in an excess of unfolded, aggregated proteins that were not degraded, thus increasing endoplasmic reticulum (ER) stress. This triggers prolonged c-Jun N-terminal kinase (JNK) signaling pathways, leading to caspase-3 activation and increase in induction od apoptosis. Another study with the ESCRT-1 protein TSG101 (known to recognize monoubiquitin or Ub_1_) in HeLa cells found it to be vital in the membrane fissure of MVB vesicle formation, eventually leading to exosome formation, proving their dual role in autophagy and exosome release [105].

## 4. Blocking Exosome Biogenesis as a Treatment of GBM

In the TME, exosomes are widely used to transport signaling molecules that, in turn, cause cancers to continue to grow and proliferate. There are, fortunately, two broad classes of exosome inhibitors, as well as others in development, for the termination of the formation of exosomes [106]. In GBM, these delivery vehicles are vital for various endogenous processes, as they are easily able to cross the BBB and last much longer in circulation than signaling molecules sent just by themselves. While there has been a large area of focus on studying the use of exosomes to deliver therapeutics, the diametrically opposite idea is to inhibit the production of exosomes to hinder the growth of many tumors, including GBM. In reference to the previous sections, we mentioned that exosomes are first made by the inward budding of the parental cell’s plasma membrane and then released once ESCRT and other proteins sort and load them with the desired cargo. These are the two main steps in exosome production, and the various proteins involved can be used as targets for inhibition of exosome biogenesis. There are many drugs already shown to inhibit exosome biogenesis; however, limited studies show their application in the treatment of GBM or even other cancers.

### 4.1. Exosome Biogenesis Inhibitors

Inhibition of the process of biogenesis of exosomes is a promising therapeutic avenue in GBM and can be initiated at the inward budding of the plasma membrane and creation of the exosome itself. Many of these inhibitors focus on disrupting the formation or the composition of the exosome plasma membrane, as failure to have these components, such as sphingomyelinase, ceramide, and cholesterol, has been shown to reduce the biogenesis of exosomes (Table 3). Of note, the small-molecule GW4869 is an inhibitor of neutral sphingomyelinase (nSMase) and has been widely studied and tested for exosome inhibition, and it has promising capabilities for inhibiting the biogenesis of unwanted exosomes and thus treating GBM. In a study looking at pediatric high-grade glioma, GW4869 proved to successfully inhibit nSMase, which converts sphingomyelin into ceramide [107]. Ceramide is important to the exosome membrane as it is used in lipid raft formations that allow exosome shedding to form the spherical shape of the exosome. This inhibition resulted in a reduction in the release of exosomes. In another study, exosomes from prostate cancer cell lines were used to show that they help promote macrophages from M1 to M2 polarization through the STAT3 signaling pathway [108]. M1 macrophages are classically activated and secrete inflammatory factors and enzymes that are used to kill pathogens and tumors, while M2 macrophages are alternatively activated, secreting tumor growth-promoting factors, vasoactive substances, and metalloproteinases, which all promote tumors. Using GW4869, the inhibition of the exosomes prevented this M2 macrophage polarization, effectively inhibiting the progression of prostate cancer cells.

Sometimes, the outcomes from some inhibitors are the side effects of the main purpose of the drug. For example, imipramine is a tricyclic antidepressant that is a serotonin and norepinephrine reuptake inhibitor. It has also been found to inhibit the activity of acid sphingomyelinase (aSMase) when it is taken into cell endosomes and lysosomes after it is protonated. The aSMase enzyme also converts sphingomyelin into ceramide through hydrolysis, and inhibition of this results in decreased MVB formation and thereby exosome secretion. While the effects of imipramine on exosomes have not been tested in GBM specifically, they have been tested in prostate cancer, showing a significant reduction in cancer cell survival times [115]. Another study showed the use of imipramine in GBM cell lines and found that it inhibited Yes-associated protein (YAP), an oncoprotein that is thought to be important in promoting growth in most tumors, including GBM, through the Hippo kinase cascade [117]. It was found that inhibition of aSMase contributes to inhibition of YAP signaling and impaired progression of GBM. Combining imipramine and TMZ showed increased synergism and further impairment of GBM progression, suggesting that combining therapeutics can result in surprising synergies.

Another class of drugs that can be used to inhibit exosome biogenesis is statins. Simvastatin is one of the most common statins that inhibits the enzyme 3-hydroxy-3-methyl glutaryl-CoA (HMG-CoA) reductase, a vital part of the synthesis of cholesterol. Simvastatin is typically used to lower low-density lipoprotein (LDL) cholesterol and to help aid in the treatment of heart disease. Since exosomes are made up of cholesterol, statins prove to be a useful tool in inhibiting exosomes. In one study, researchers used irradiated human GBM U87MG cells to understand the role of exosomes and the inhibitory properties of simvastatin [112]. They found that upon radiation, these GBM cells had an increase in exosome release, showing that they are heavily involved in the radiation therapy resistance that tumor cells gain. Treatment with simvastatin and heparin resulted in an overall decrease in exosome release and proliferation. Statins have also recently been looked at for cancer treatments as they exhibit other anti-tumor effects, including inhibition of angiogenesis, cell cycle arrest at G1 phase, reduction in activity of MAPKs, induction of apoptosis, and many more [118]. An observational study comparing GBM patients who took any type of statin and patients without it showed that those who had not been treated with statins had worse median survival rates compared to those who were treated [119]. Preclinical GBM cell cultures and xenograft mouse models were also looked at following treatment with simvastatin, which showed a reduction in tumor size and weight, and an increase in apoptosis [120,121,122]. These studies once again show the dual role some drugs may have and how they can be repurposed to counter tumor therapy resistance from exosomes.

Spiroepoxide and DPTIP (2,6-dimethoxy-4-(5-phenyl-4-thiophen-2-yl-1H-imidazole-2-yl)-phenol) are two other nSMase inhibitors that both show effects of exosome inhibition, with DPTIP being the most potent of the two [123]. Other common exosome biogenesis inhibitors include pantethine (co-enzyme A inhibitor), indomethacin (COX-1 and COX-2 inhibitor), and glibenclamide (ATP-sensitive potassium channel inhibitor) [106]. These drugs have yet to be applied to the treatment of GBM; however, they show promising results in other cancer models and can be potential avenues for exosome inhibition in GBM as well.

### 4.2. Exosome Release Inhibitors

Release of exosomes occurs when the MVBs fuse with the plasma membrane and release all the ILVs inside. This process is a complex mechanism that is dependent on the interaction between actin and microtubules for transport, Rab proteins from the GTPase family, Ca^2+^ channels, SNARE proteins, and ESCCRT-dependent pathways. Thus, a variety of targets offer many avenues for drugs to inhibit certain mechanisms to prevent exosome release. One drug that has been studied extensively is calpeptin, a reversible inhibitor of caplain, which is a Ca^2+^-dependent cysteine protease that mainly exists as calpain-1 and calpain-2 [124,125]. In cancers such as GBM, caplain is dysregulated and has varying effects such as increased cell proliferation, induction of apoptosis, and tumor migration. Since it works for cleaving many different proteins, many diverse effects have been found following calpain-mediated degradation of proteins in the cells. For example, caplain hydrolyzes filamin A into a 90 kDa carboxyl fragment (ABP90), a cytoskeletal protein that assists in cell shape and migration. The treatment of calpeptin in GBM cells surprisingly promoted the proliferation and invasion of GBM and decreased the efficacy of TMZ treatment [126]. Alternatively, another study showed that knockdown of caplain-2 resulted in increased apoptosis after TMZ administration [127]. These studies show that inhibition of caplain, with or without calpeptin, can have varying results. In the case of prostate cancer, the use of calpeptin to inhibit the secretion of microvesicles was used to overcome the resistance formed to docetaxel, a small-molecule reversible inhibitor of microtubulin. Once calpeptin was administered, microvesicle levels were noticeably lower, allowing docetaxel to accumulate in the cell and increase levels of apoptosis, decrease tumor volume, decrease vascularization, and decrease cell proliferation [128]. With the previous studies performed in GBM models, calpeptin shows promise as an inhibitor of exosome release and may be useful for the treatment of GBM.

### 4.3. Other Exosome Inhibitors

Previous studies of exosome formation and release have shown immense importance in maintaining an acidic pH within the cell and the TME itself. In one study, it was proven that inhibition with the use of proton pump inhibitors (PPIs) greatly reduced the ability of exosomes in melanoma cells to be trafficked out of the cell and membrane fusion [129]. Since then, other studies have looked at various models to confirm the effects of PPIs on exosome formation and have been used as a possible therapy. For example, one study showed the effects of PPIs in the context of hepatotoxicity. It was found that tumor-derived exosomes showed greater ability to induce fatty liver from activated macrophages producing tumor necrosis factor (TNF), ultimately increasing the toxicity of chemotherapies. In this study, investigators utilized PPIs to inhibit exosome hepatocellular uptake to prevent the activation of macrophages producing TNF through enhanced macropinocytosis. Macropinocytosis is a core endocytic pathway through which cells internalize large volumes of extracellular fluid and solutes in a non-selective manner. This process supports diverse physiological functions, but it can also be involved in pathological contexts, contributing to disease progression. Increased uptake with the use of the PPI Rabeprazole led to increased clearance and reduced liver macrophage activation [130]. Another study looking at precancerous lesions in rats utilized the PPI Pantoprazole [131]. These lesions were first induced by administration of diethylnitrosamine and 2-acetylaminoflueorene, which showed elevated levels of exosomes. Administration of Pantoprazole caused a significant decrease in levels of exosomes in the rat model.

## 5. Engineering of Exosomes for Use as Vehicles for Drug Delivery to GBM

While a majority of this article has discussed the detrimental effects exosomes pose to the body by promoting the proliferation and survivability of GBM tumors, extensive research has also been performed to utilize the properties of exosomes as a means of drug delivery [132,133,134]. Because these are endogenously produced, they contain a variety of cell surface proteins such as tetraspanins (e.g., CD9, CD37, CD53, CD63, CD81, CD82, and lysosomal associated membrane protein 2 isoform B or LAMP2B), antigen-presenting molecules such as of major histocompatibility complex I (MHC I) and MHC II, adhesion molecules (integrins α/β, P-selectin, intercellular adhesion molecules or ICAMs), signaling receptors such as FasL (Fas Ligand), TNFR (Tumor Necrosis Factor Receptor), TFR (Thyroid Follicular Receptor), PD1 (Programmed Death 1), and PD-L1 (Programmed Death Ligand-1), and a variety of lipids and glycoproteins [135]. These provide exosomes with the ability to evade elimination by the immune system and be able to deliver various cargoes, as discussed before, that contribute to therapy resistance. Additionally, surface molecules allow them for endocytosis (vesicular membrane allowing transport of substances inside from the external environment) and transcytosis (vesicular transport of macromolecules from one side of a cell to the other) through the tricky BBB made of endothelial cells. Crossing the BBB has been a major hurdle to overcome for many GBM therapeutics, making the potential for exosomes as the next mode of drug delivery a high possibility. Many studies utilizing exosomes as therapeutics derive them from the tissue that is meant to be targeted; this helps targeted delivery to occur, as they already have the parent cell markers and target markers. Many exogenous efforts have also been made to tinker or engineer exosomes for tissue-specific targeting by adding or removing surface proteins during the drug loading process. Since exosomes already contain a variety of cargo types (DNA, RNA, proteins, lipids, etc.), encapsulation of new cargo (the drug of choice) will secure the cargo and prevent it from being degraded or washed out [135]. These properties show the vast reasons why exosomes have a high potential to be developed as drug delivery vehicles and why much work has been performed to incorporate drugs that were previously not viable to become effective. Most exosomes are purified using ultracentrifugation and loaded with the cargo of choice using co-incubation. Some current studies utilized engineered therapeutic exosomes or EVs as drug delivery vehicles for the treatment of GBM (Table 4).

### 5.1. Exosomes for Delivery of Inhibitors of Angiogenesis to GBM

**Figure 4:**
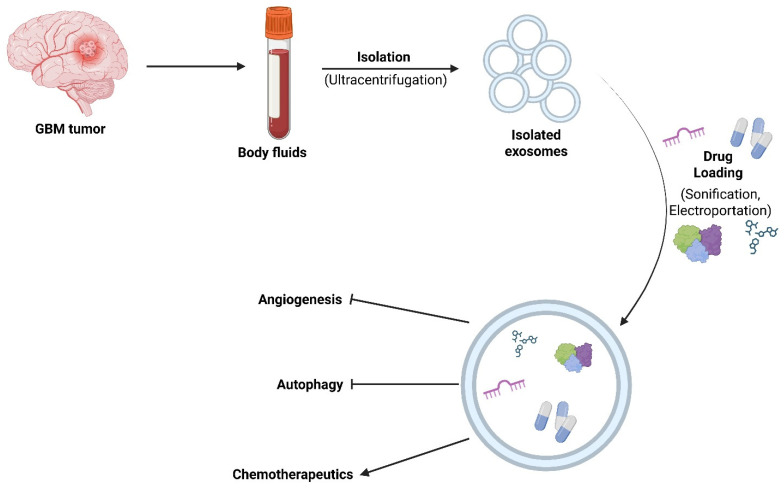
Schematic presentation of the drug loading process in exosomes for delivery of therapy to GBM. Extraction of exosomes for use in GBM treatment does not have to be specifically derived from GBM tumors; however, these exosomes may be more effective since they already have similar markers that GBM tumors can recognize. Loading drugs into exosomes greatly increases their effectiveness due to the enhanced ability to cross the BBB.

Exosomes can be isolated, purified, and engineered with therapeutic cargoes for inhibiting the growth of GBM (Figure 4). As discussed before, induction of angiogenesis is a vital method of therapeutic resistance that promotes aggressive tumor growth. The hypoxic TME promotes the release of exosomes with pro-angiogenic factors [146]. Therefore, delivery of anti-angiogenic factors to the tumor has been an area of immense interest. Like other GBM therapies, current drugs are not effective in crossing the BBB and staying long enough in circulation to be effective in preventing angiogenesis. Studies utilizing exosomes for drug delivery to GBM tumor models have shown a decrease in angiogenesis as well as an increase in inducing apoptosis. One study used exosomes derived from murine mesenchymal stem cells with the macrolide (protein synthesis inhibitor) Rapamycin (Rapa) in U87MG cells [145]. The investigators compared the effectiveness of Rapa when delivered by itself or within the exosomes and found significant BBB penetration with the exosomes, with higher concentrations of Rapa at the tumor site. Rapa was then able to induce a host of effects, including decreased levels of IL-6, TNF-α, and VEGF (prominent pro-angiogenic factors) and increased cell cycle arrest, inhibition of cell proliferation, and decreased tumor size. Another study utilized milk-derived exosomes loaded with mithramycin (Mit-A) in human GBM U87MG and LN229 cells [142]. Mit-A binds to GC-rich sequences in DNA, preventing transcription factors from binding to gene promotor regions. This leads to anti-tumor activity; however, Mit-A has severe systemic hepatotoxicity and other side effects that limit its use. When Mit-A was delivered using exosomes, the hepatotoxicity was effectively mitigated. The blockage of Specificity Protein 1 (SP1) transcription factor binding resulted in decreased VEGF activation and decreased angiogenesis. It also decreased proliferation, migration, and induced apoptosis. These show how valuable exosomes can be for drug delivery for GBM therapy.

### 5.2. Exosomes for Delivery of Autophagy Inhibitors to GBM

Another major mechanism of therapeutic resistance to GBM therapies is activation of the autophagic pathway. This pathway is induced, unfortunately, by TMZ and other prominent chemotherapeutic drugs used for the treatment of GBM, in addition to GBM-derived exosomes. The role of autophagy has proven to be vital in the survivability of GBM tumors, and targeting this mechanism has high potential to provide effective treatment [86,88,147]. Many of the autophagy inhibitors target STAT3. In general, the role of STAT3 in autophagy is highly context dependent. In non-tumor pathological conditions, such as ischemic stroke, STAT3 activation has been shown to suppress autophagy through down regulation of Beclin-1. In this setting, small extracellular vesicles (sEVs) derived from human induced pluripotent stem cell–derived mesenchymal stem cells promote STAT3 signaling, leading to inhibition of ischemia-induced autophagy [148]. In contrast, in the context of cancers including GBM, STAT3 signaling is generally associated with promotion of autophagy through its downstream survival and stress-response pathways. For example, exosomes loaded with anti-STAT3 siRNA in the human GBM U87MG cell line showed decreased autophagy and increased apoptosis. When compared to treatment with just anti-STAT3 siRNA, loaded exosomes showed greater BBB penetration, prolonged mean survival time (MST), greater STAT3 silencing, and negligible side effects on major organs [137]. Another study used HEK293T-derived exosomes loaded with miRNA-124 in human GBM U373MG cells [139]. miRNA-124 is typically downregulated in GBM tissues as it leads to inhibition of proliferation, migration, and EMT. Upon delivery of miR-124 to GBM cells, total STAT3 was found to be reduced, and the activation of STAT3 by phosphorylation at the Tyr705 and Ser727 residues was inhibited, eventually leading to decrease in autophagy. Increased miRNA-124 also caused downregulation of TGFβ and IL-10 and upregulation of IL-6 to promote M1 macrophage polarization, leading to GBM growth inhibition.

### 5.3. Exosomes for Delivering Chemotherapeutic Drugs to GBM for Induction of Apoptosis

Many chemotherapeutic drugs are currently not effective in GBM treatment because of their inability to cross the BBB. As we have seen in other drug delivery methods, exosomes provide an effective way of crossing the BBB and delivering drugs with targeted treatment. One common chemotherapeutic is doxorubicin (DOX). DOX has a host of mechanisms that inhibit cell growth, such as DNA intercalation, topoisomerase II inhibition, reactive oxygen species (ROS) generation, and membrane disruption, eventually leading to cell death [149]. In one study, the proton-coupled oligopeptide transporter 2 (PepT2) was engineered into the hydrophilic core of exosomes from mouse BV2 microglial cells [136]. PepT2 contains cysteine residues that cross-link and form disulfide bonds to DOX, stabilizing it inside the exosome during the loading process. Once the target is reached, glutathione (GSH) cleaves the disulfide bonds, and DOX is released for use. Utilizing these engineered PepT2 exosomes on human GBM U87MG cells and orthotopic mouse models showed efficient BBB penetration and the anti-tumor activity of DOX with no obvious toxicity to the major organs (e.g., liver, spleen, kidney, and heart) [136]. Another study loaded DOX and Cetuximab (CTX) into rat GBM C6 cell-derived exosomes, with engineered polyethylene glycol (PEG) lipids for CTX binding [140]. CTX is an anti-EGFR antibody, which effectively blocks EGF binding to its receptor (EGFR) and activation of downstream kinases. Administration of these exosomes in GBM model in rats showed two times the BBB penetration compared to just CTX and DOX alone, with no morphological changes or lesions to major organs. This led to inhibition of EGFR and increased expression of Bax and caspase-3, which are acclaimed biomarkers of apoptotic cell death. Paclitaxel (PTX) has been loaded into human GBM U87MG cell-derived exosomes and showed significantly lower levels of growth in U87MG cells when compared to PTX alone [143].

Although TMZ can cross the BBB (relative to the other chemotherapeutics), it produces other methods of resistance due to its ability to activate autophagy. The idea of administering TMZ and an autophagy inhibitor can have the potential to reverse the therapeutic resistance that TMZ alone induces. One study utilized exosomes with folic acid conjugated on the surface with TMZ and quercetin (QCT) [144]. QCT, like DOX, has a host of effects such as topoisomerase II inhibition and ROS generation, and other anti-inflammatory effects. In addition to those, it is also involved in the induction of regulated autophagy and apoptosis. In this case, QCT suppressed the PI3K/Akt/mTOR survival pathway. Delivery of these exosomes resulted in increased apoptosis and reduced angiogenesis, showing that the co-delivery of drugs is a promising avenue for the treatment of cancers, especially using exosomes. Previous studies confirmed that a synergistic combination of two different therapeutic agents was significantly effective in inhibiting therapy resistance mechanisms such as angiogenesis and autophagy, leading to induction of apoptotic signaling in GBM in cell culture models [150,151] as well as in animal models [152,153]. Use of the engineered exosomes is expected to further enhance the efficacy of the synergistic combination therapy in GBM in preclinical animal models, eventually paving the path for translation of this approach to clinical settings.

## 6. Conclusions and Future Directions

Exosomes play a significant role in the aggressive biology and therapy resistance of GBM. By transporting growth factors, receptors, and regulatory miRNAs across the tumor microenvironment, they enhance angiogenesis, suppress immune activity, and promote survival pathways such as autophagy. These functions, combined with the intrinsic resistance of GBM to conventional chemotherapeutics and the restrictive nature of the BBB, emphasize the importance of exosome-mediated communication as a major barrier to effective treatment. Targeting exosome biogenesis and release represents a promising therapeutic avenue, particularly because of the mechanistic overlaps between exosome generation and autophagy pathways.

Controlling engineering of therapeutic exosomes is an alternative avenue in the treatment of GBM. A major translational challenge, however, is the lack of standardized methods for exosome isolation, characterization, and quality control, which leads to large variability in vesicle purity, concentration, and functional assessment. Recent analyses of human exosome studies highlight substantial inconsistencies across platforms such as ultracentrifugation, precipitation, size-exclusion chromatography, and microfluidics, all of which strongly influence yield, cargo composition, and particle integrity. Similar calls for rigorous standardization emphasize the need for reproducible protocols in density-gradient ultracentrifugation, RNA profiling, and downstream analytics to support clinical translation and regulatory acceptance of exosome-based therapeutics for treatment of GBM. Due to the lack of widely adopted workflows with good manufacturing practices (GMP), it remains difficult to compare findings across laboratories or generate clinically scalable exosome products [154].

Another critical dimension involves the ability of exosomes to traverse the BBB. Preclinical data indicate that exosomes—due to their size (30–150 nm) and endogenous membrane composition—can cross the BBB under both physiological and pathological conditions, though the molecular mechanisms remain poorly defined. Their natural capacity to penetrate the BBB has already been exploited experimentally to deliver siRNAs, drugs, and metabolic modulators into GBM tissue, highlighting their potential to bypass one of the most formidable obstacles in neuro-oncology drug delivery. This unique property positions exosomes as promising vectors for therapeutics capable of reaching infiltrative GBM regions that are inaccessible to most conventional agents [155].

At the same time, regulatory constraints in the United States sharply limit clinical deployment of exosome-based interventions in GBM. According to the FDA guidance and notices, no exosome products are currently approved for therapeutic use, regardless of indication or route of administration. However, recent regulatory milestones underscore the growing clinical readiness of exosome-based therapeutics. In 2025, the FDA granted multiple Orphan Drug Designations (ODDs) for exosome-based treatments targeting GBM, including the Exousia Pro-manufactured exosome-mediated nucleic-acid delivery platform, which was recognized for its potential to enhance therapeutic penetration across the BBB and improve treatment outcomes in GBM. All 2025 ODDs for exosomes were supported by rigorous preclinical studies using human-derived GBM xenografts, signaling regulatory acknowledgement of exosomes as emerging, clinically relevant agents in neuro-oncology drug development [156].

Despite different hurdles, the therapeutic promise of exosomes remains substantial. Their intrinsic ability to cross the BBB and selectively accumulate in tumor tissue provides a powerful platform for delivering combination chemotherapeutics, RNA therapeutics, or targeted inhibitors to kill GBM and, most importantly, GSC cells in the tumor while reducing systemic toxicity [143,144,145,157,158]. Preclinical work using engineered or cargo-loaded exosomes demonstrates significant potential, although major refinements in loading efficiency, targeting specificity, vesicle stability, and manufacturing reproducibility are still required [159,160].

Future studies should integrate these complementary themes: systematic evaluation of exosome biogenesis and release inhibitors in GBM models, with emphasis on intersecting autophagy and vesicle-trafficking pathways; mechanistic dissection of the autophagy–exosome interface, including the shared dependency on endosomal maturation and amphisome formation; and continued refinement of exosome-based delivery systems, guided by standardized isolation protocols, validated analytical benchmarks, and adherence to GMP-compliant frameworks.

Overall, combining exosome-targeted inhibition with exosome-enabled drug delivery may provide a dual strategy capable of overcoming current resistance mechanisms and improving therapeutic outcomes for GBM.

## Figures and Tables

**Table 1:** Commonly found miRNAs in GBM and their effects in promoting therapy resistance.

miRNA	Role and Expression	Target(s)	Impact of miRNA in GBM	Results of miRNA Alteration	Reference
miR-130a-3p	TS-miR and decreased	Cytoplasmic polyadenylation element binding protein 4 (CPEB4)	Increased CPEB4 caused GBM growth and migration	Increased miR-130a-3p inhibited proliferation, migration, and EMT, while increased sensitivity to TMZ	[70]
miR-7	TS-miR and decreased	Insulin receptor substrate (IRS)-1 and IRS-2	Increased IRS-1 and IRS-2 proteins promoting Akt pathway and growth	miRNA-7 and erlotinib synergistically inhibited cell survival and augmented apoptotic death in U373MG cells	[71]
miR-145	TS-miR and decreased	A Disintegrin And Metalloprotease 17 (ADAM17)	Upregulated ADAM17 correlating with GBM growth and TMZ resistance	Increased miR-145 inhibited ADAM17 and enhanced TMZ sensitivity	[72]
miR-21	Onco-miR and increased	Programmed cell death protein 4 (PDCD4) and human MutS homolog 2 (hMSH2)	Suppressed PDCD4 and hMSH2 contributing to radiation resistance	miR-21 knockdown increased PDCD4 and hMSH2, which contributed to apoptosis and G_2_ arrest of T98G cells	[73]
miR-21	Onco-miR and increased	PTEN (phosphatase and tensin homolog), PDCD4, RECK (reversion-inducing cysteine-rich protein with Kazal motifs), and STAT3 (signal transducer activator of transcription 3)	Increased VEGF binding to VEGFR2 for angiogenesis	Decreased miR-21 caused decreases in angiogenesis and cell proliferation in GBM	[74,75]
miR-200a	TS-miR and deceased	MGMT	Inhibits MGMT activity	Increasing its expression inhibits MGMT and reverses TMZ resistance	[76]
miR-374b-3p	Onco-miR	PTEN	Enhances tumor angiogenesis by inducing M2 polarization of tumor-associated macrophages (TAMs)	Targeting miR-374b-3p expression may serve as a potential therapy against angiogenesis in GBM and GSCs	[77]
miR-221	Onco-miR and increased	Dynamin 3 (DNM3)	Causes tumor progression and TMZ resistance	RELA increases miR-221, which decreases DNM3 and tumor growth, but inhibition of miR-221 decreased cell proliferation, migration, and TMZ resistance	[78]
miR-9	Onco-miR and increased	Collagen type XVIII alpha 1 chain (COL18A1), thrombospondin 2 (THBS2), patched 1 (PTCH1) and egl-9 family hypoxia inducible factor 3 (PHD3)	Causes MDR1overexpression and chemoresistance	Delivery of anti-miR-9 to the resistant GBM cells reverses the expression of MDR1 and sensitizes GBM cells to TMZ	[79,80]
miR-1238	Onco-miR and increased	Caveolin-1 (CAV1)	Increases EGFR and PI3k/Akt/mTOR pathways	Inhibition of miR-1238 leads to increased levels of CAV1 and caspase-3 due to chemosensitivity to TMZ	[81]
miR-151a	TS-miR and decreased	X-ray repair cross-complementing protein 4 (XRCC4)	Promotes XRCC4-mediated DNA repair and TMZ resistance	Restored miR-151a expression sensitizes TMZ-resistant GBM cells via inhibiting XRCC4-mediated DNA repair	[82]
miR-25-3p	Onco-miR and increased	F-box and WD repeat domain-containing-7 (FBXW7)	Promoted c-Myc and cyclin E expression by downregulating FBXW7	Overexpression of miR-25-3p facilitates cell proliferation and TMZ resistance of sensitive GBM cells	[83]

**Table 2:** Intersection of exosome and autophagy mechanisms in developing therapy resistance in GBM and other cancer models.

Cancer Model	Exosomes	Mechanisms	Results	Reference
GBM	GSCs derived programmed death-ligand 1 containing exosomes (PD-L1-Exos)	PD-L1-Exos activated AMPK/ULK1 pathway mediated protective autophagy	Enhanced TMZ resistance in GBM in vitro and in vivo	[91]
Glioma	Hypoxic glioma-derived exosomes (HGD-Exos)	HGD-Exos markedly facilitated autophagy and M2-like macrophage polarization	M2-like macrophage polarization occurred via the IL-6-pSTAT3-miR-155-3p-autophagy-pSTAT3 positive feedback loop, likely causing immunosuppressive microenvironment	[92]
Hepatitis B virus (HBV) associated liver cancer	Exosomes from HBV-infected liver cancer cells	HBV-associated liver cancer exosomes activated CMA pathway	Exosomes from HBV-infected liver cancer cells decrease apoptosis when treated with oxaliplatin	[93]
Patients with non-small cell lung cancer (NSCLC)	Circulating exosomal miR-425-3p from NSCLC patients	Cisplatin induced c-Myc to bind to exosomal miR-425-3p promoter and transactivated its expression, facilitating autophagy activation	Exosomal miR-425-3p facilitated autophagic activation in the recipient cells by targeting AKT1, eventually leading to chemoresistance	[94]
Lung adenocarcinoma (LUAD) cells	Exosomes containing long noncoding RNA (lncRNA) small nucleolar RNA host gene 7 (SNHG7) from docetaxel-resistant LUAD cells	SNHG7 promoted autophagy, activated PI3K/AKT pathway to promote M2 macrophage polarization to induce ubiquitination and degradation of PTEN	Exosomal SNHG7 transmitted from docetaxel-resistant LUAD cells to parental LUAD cells enabled docetaxel resistance	[95]

**Table 3:** Inhibitors of exosome biogenesis and release for treatment of GBM and other cancers.

Inhibitor	Inhibition of Exosomal Process	Inhibition of Molecule or Process	Effects of Inhibition	Results in Cancer	Cancer	Reference
TAK981	Biogenesis	Inhibition of UMOylation of hnRNP A2/B1 inhibited the exosome-sorting process of miR-204-3p	Inhibition of miR-204-3p blocked tube formation of vascular endothelial cells	Inhibited angiogenesis and tumor growth	GBM	[109]
NSC23766	Biogenesis	Inhibition of exosome-derived RAC1 activation	Inhibition of RAC1 inhibited AKT activation and NRF2 nuclear translocation	Inhibition of RAC1/AKT/NRF2 pathway inhibited M2 polarization of microglia	GBM	[110]
Short hairpin RNA (shRNA)	Release	sh Ras-associated protein 27a (shRab27a) knocked down Rab27a mRNA	Inhibition of docking of MVBs to plasma membrane	Decreased release of small EVs and growth in GL261 cells	GBM	[111]
GW4869	Biogenesis	Inhibition of the enzyme neutral sphingomyelinase (nSMase)	Inhibition of sSMase blocked ceramide production for lipid membrane	Inhibition of secretion of exosomes impaired cell motility (migration and invasion)	Pediatric-type diffuse high-grade gliomas (PDHGG)	[107]
Simvastatin and Heparin	Biogenesis	Inhibition of HMG-CoA reductase	Inhibited radiation derived exosome uptake in recipient cells	Inhibition of radiation derived exosome uptake inhibited cell proliferation and survival	GBM	[112]
Glibenclamide	Biogenesis	Inhibition of sulfonylurea receptor 1 (SUR1)	Inhibition of SUR1 blocked KH-type splicing regulatory protein (KHSRP) phosphorylation	SUR1-inhibited exosomes impaired tumor growth and CAF (cancer associated fibroblasts) accumulation	Non-small cell lung carcinoma (NSCLC)	[113]
Indomethacin	Biogenesis	Inhibited cyclooxygenases (COX-1 and COX-2)	Repression of exosomal ATP-transporter A3 (ABCA3) expression	Depletion of ABCA3 augmented subcellular accumulation and prolonged nuclear retention of cytotoxic drugs (doxorubicin and pixantrone)	Diffuse large B-cell lymphoma (DLBCL)	[114]
GW4869	Biogenesis	Impeded macrophages from differentiating into M2 cells	Inhibition of Akt and STAT3 signaling pathways	Inhibition of M2 differentiation inhibited tumor growth	Prostate cancer	[108]
Imipramine	Biogenesis	Inhibition of acid spingomyelinase (aSMase)	Blocked exosome biogenesis	Sensitized the resistant cancer cells to chemotherapy	Prostate cancer	[115]
Chloramidine and bisindolylmaleimide-I	Release	PKC	Blocked externalization of phosphatidylserine	Enhanced efficacy of chemotherapeutic drug-mediated apoptosis	Prostate cancer	[116]

**Table 4:** Engineered exosomes or EVs for enhanced therapeutic delivery and efficacy in GBM.

Source of Exosomes (Exos) or EVs	Purification of Exos or EVs	Drug	Drug Loading into Exos or EVs	Engineered Exos or EVs Delivery to GBM Model	Mechanisms of Drug Action	Results Showing Drug Efficacy	Reference
Oligopeptide-modified Exos (Pep2-Exos) derived from BV2 mouse microglia	Ultrafiltration	Doxorubicin (DOX)	Co-incubation	U87MG cells xenotransplanted in brains of BALB/c nude mice to establish orthotopic model	Pep2 cysteine residues cross-link to DOX in hydrophilic core of exosome but GSH breaks these bonds once the target is reached and DOX is released	Intravenous injection of Pep2-Exos-DOX in orthotopic mouse model resulted in efficient BBB penetration for efficacy and no obvious toxicity against liver, spleen, kidney, and heart	[136]
Exos from human leukemia monocytic cell line THP-1	Ultrafiltration	Anti-STAT3 siRNA	Co-incubation	Orthotopic U87MG xenografts	Exos loaded with Angiopep-2 (An2) functionalized STAT3 and siRNA (Exos-An2-siRNA) blocks STAT3 activation	Efficient STAT3 silencing increased apoptosis in orthotopic U87MG xenografts with limited side effects and significant enhancement of median survival time (MST)	[137]
HEK293T-derived EVs	Ultrafiltration	Cytosine deaminase (CD) fused to uracil phosphoribosyltransferase (UPRT)	Co-incubation	U87MG implanted into the flanks of nude SCID mice	Therapeutic CD-UPRT-EVs convert 5-fluorocytosine to 5-fluorouracil	Therapeutic CD-UPRT induced defective DNA replication and apoptosis	[138]
HEK293T-derived EVs	ExoQuick method and ultrafiltration	miRNA-124	Lipofecion and co-incubation	U373MG cells and microglia co-culture in a 3D microfluidic system	miR-124-EVsdecreased mRNA levels of tumor progression and markers of M2 microglial polarization	miR-124 EVs suppressed mRNA levels of tumor progression and M2 microglial polarization markers	[139]
Exos from rat GBM C6 cells	Ultracentrifugation	Cetuximab (CTX) in combination with doxorubicin (DOX)	Post-insertion method	C6 model in rats	CTX-Exo-DOX crossed BBB and significantly decreased Bcl-2 and increased Bax and cleaved caspase-3 for apoptosis in GBM cells	CTX-Exo-DOX significantly inhibited proliferation of tumor cells and prolonged survival time of tumor-bearing rats	[140,141]
Milk-derived (mEVs)	3000 polyethylene glycol (PEG) precipitation method	Mithramycin (Mit-A)	Freeze–thaw method	U87MG and LN229 cells	Mit-A shows anti-tumor activity by binding to GC rich sequences of DNA, blocking SP family transcription factors to gene promoters, but it causes systemic hepatotoxicity and other side effects	The mEV (Mit-A) formulation transported Mit-A more effectively than free Mit-A, significantly inhibited glioma cell growth, and migration, and induced apoptosis due to inhibited SP1 pathway that could increase Myc, P21, and VEGF	[142]
U87MG derived Exos	Centrifugation	Paclitaxel (PTX)	Two methods (incubating and sonication)	U87MG cells	PTX acts as a mitotic inhibitor (microtubule stabilizing agent) for anti-cancer effects, but cannot pass through BBB	PTX-Exos significantly inhibited cell growth compared to free PTX	[143]
Exos conjugated with folic acid (FA)	Ultracentrifugation	Temozolomide (TMZ) and quercetin (QCT)	Sonication-assisted method	U87MG and U251MG in vitro and in vivo	Combination of drugs inhibited the PI3K/Akt/mTOR pathway	TMZ-QCT-Exos-FA caused significant tumor size reduction, increased apoptosis, reduced angiogenesis	[144]
Exos from murine mesenchymal stem cells (MSCs)	Ultracentrifugation	Rapamycin (Rapa)	Real-time incubation method	U87MG cells	Exo-encapsulated Rapa (Exo-Rapa) inhibited elongation of protein synthesis	Exo-Rapa enhanced BBB penetration, increased cell cycle arrest, inhibited cell proliferation, decreased tumor size, and reduced angiogenesis	[145]

## Data Availability

No new data were created or analyzed in this study. Data sharing is not applicable to this article.
